# Continuous-flow DNP polarizer for MRI applications at 1.5 T

**DOI:** 10.1038/srep44010

**Published:** 2017-03-14

**Authors:** V. Denysenkov, M. Terekhov, R. Maeder, S. Fischer, S. Zangos, T. Vogl, T. F. Prisner

**Affiliations:** 1 Institute of Physical and Theoretical Chemistry and Center for Biomolecular Magnetic Resonance, Goethe University, Frankfurt am Main, Germany; 2 Comprehensive Heart Failure Center, University Hospital Würzburg, Würzburg, Germany; 3 Institute of Diagnostic and Interventional Radiology, University Hospital Frankfurt, Frankfurt am Main, Germany

## Abstract

Here we describe a new hyperpolarization approach for magnetic resonance imaging applications at 1.5 T. Proton signal enhancements of more than 20 were achieved with a newly designed multimode microwave resonator situated inside the bore of the imager and used for Overhauser dynamic nuclear polarization of the water proton signal. Different from other approaches in our setup the hyperpolarization is achieved continuously by liquid water flowing through the polarizer under continuous microwave excitation. With an available flow rate of up to 1.5 ml/min, which should be high enough for DNP MR angiography applications in small animals like mice and rats. The hyperpolarized liquid cooled to physiological temperature can be routed by a mechanical switch to a quartz capillary for injection into the blood vessels of the target object. This new approach allows hyperpolarization of protons without the need of an additional magnet and avoids the losses arising from the transfer of the hyperpolarized solution between magnets. The signal-to-noise improvement of this method is demonstrated on two- and three-dimensional phantoms of blood vessels.

Magnetic resonance imaging (MRI) techniques allow non-invasive visualization of living tissue morphology and functions with a high soft tissue contrast. Although these techniques are for many years routinely used in clinical practice, technological, chemical and methodological research focuses on the improvement of the intrinsically low sensitivity of the method arising from the small polarization of the nuclear spin signal. Improvements could be achieved from elevated static magnetic field strengths, which increase the nuclear spin polarization and detection sensitivity. This opened up many new avenues and application fields of the method. On the other hand, moving the technology to larger field values also increases the size and the costs of an MRI scanner. Much improvement has also been obtained by employing contrast agents, mostly based on Gadolinium complexes. However, the administration of Gadolinium-based contrast agents is associated in rare cases with the risk of allergic reactions or development of a systemic nephrogenic fibrosis^1^,^2^. Current research even showed that some types of Gadolinium-based MRI contrast agents remain in the brain - with unknown long-term effects^3^,^4^. Besides, once being applied the contrast agents stay in the blood pool and kidney for several hours making it impossible to repeat experiments under the same conditions within this time.

An alternative way of increasing the NMR signal is to create nuclear spin magnetization higher than the Boltzmann polarization at thermal equilibrium at a given static magnetic field. Such hyperpolarization methods have been explored extensively for NMR spectroscopy and MRI. Hyperpolarized noble gases (^3^He and ^129^Xe) by optical pumping have been used for morphological and functional MRI studies of the lung^5^,^6^,^7^. Other hyperpolarization techniques developed for use in MRI are para-hydrogen-induced polarization (PHIP) and dynamic nuclear polarization (DNP). PHIP relies on the transfer of spin order from para-hydrogen to proton nuclei in the targeted molecule during catalysed chemical reactions. First applied for NMR spectroscopy^8^,^9^, PHIP has since been refined and extended to a variety of chemical substances and used for signal enhancement in MRI both in a direct or indirect (SABRE: signal amplification by reversible exchange) way^10^. DNP accomplishes the polarization transfer from unpaired electron spins of radicals to the nuclear spins of the target molecules by microwave irradiation of forbidden or allowed electron spin transitions^11^. The first method (solid effect or cross-effect) is used for solids whereas the second method (Overhauser DNP) is used mostly for samples in the liquid state.

Currently, dissolution DNP is the only commercially available hyperpolarization method of liquids for MRI^12^,^13^. It uses metabolites such as ^13^C-pyruvate, which are polarized external to the MRI magnet at very low temperatures (~1 K) by microwave irradiation. Within 35 minutes a nuclear polarization of 36% and within 5 minutes a polarization of 10% has been achieved^14^. After that, the sample is rapidly dissolved in hot liquid, transported to the imager magnet and injected into the body. The typical dissolution and transfer time of 5–15 seconds limits the *in*-*vivo* usage to ^13^C. Therefore, the method is mostly used for the study of cellular metabolism with pyruvate, lactate or other metabolic precursors labelled with low-gyromagnetic ratio nuclei with long relaxation times^15^. The usage of ^1^H as imaging nuclei for dissolution DNP has been once demonstrated^16^, however, it is not a wide-spread approach due to the short relaxation times and loss of proton polarization within the transfer step.

In contrary, hyperpolarization by the Overhauser mechanism is well suited for physiological solutions under continuous flow condition because of the rapid polarization transfer from the unpaired electrons of the radicals to proton spins of the solvent. The polarization transfer in liquids works most efficiently at low magnetic fields. First Overhauser MRI^17^,^18^, and PEDRI *in*-*vivo* experiments^19^ were demonstrated at 6–15 mT with EPR excitation of the whole mouse/rat body. Such experiments however cannot be performed in clinical MRI scanners at higher magnetic fields due to the absorption and heating of higher frequency microwaves in living tissues.

Therefore most Overhauser DNP experiments for NMR spectroscopy and MRI applications have been realized at a polarizing magnetic field of approximately 0.35 T, corresponding to an EPR frequency of approximately 9.8 GHz (“X-band”). Because this magnetic field is lower than the field used for most NMR and MRI applications a second magnet, either nearby^20^ or incorporated into the fringe field of the MRI magnet^21^,^22^ have been used for the DNP polarization process. At low magnetic fields, the incorporation of a compensation coil into the MRI scanner for the polarization transfer step has been explored^23^. However, in all these cases, the polarization achieved at the imaging site is downscaled by the ratio between the polarizing and the imaging field. In the case of an X-band polarizer (0.3 T) and 1.5 T MRI scanner this reduction factor is roughly 1/5.

We decided to explore another approach by using the same magnetic field both for the DNP polarization process and for MRI detection. This “in-bore” Overhauser-DNP approach is free of the Boltzmann field ratio penalty and other polarization losses resulting from a long transfer path from the polarizer to the imaging object^24^. As DNP agent the nitroxide radical TEMPOL (4-hydroxy-2,2,6,6-tetramethylpiperidine-1-oxyl, Sigma-Aldrich, USA) was used to polarize the water protons by microwave excitation at 42 GHz. To avoid strong heating of the liquid TEMPOL/water solution, the microwave excitation has to be performed in a microwave resonance structure, which minimizes the exposure of the flowing liquid to the electrical microwave field component. This, on the other hand reduces the possible sample size and flow rate. Here we report on our latest improvements of such an in-bore Overhauser-DNP polarizer by using a new multimode resonator (exploiting the TE_013_ mode instead of the fundamental TE_011_ mode). With a more powerful microwave amplifier 3-fold larger flow rates with 2-fold higher DNP signal enhancement has been achieved in comparison to our previous design^24^. Together with other implementations, such as a switch routing the hyperpolarized water solution either to the imaging object or to a reservoir, a temperature and pressure sensor and a heat-exchanger to keep the hyperpolarized water solution at physiological temperatures, our in-bore DNP polarizer is heads towards *in*-*vivo* studies on animals. The polarizer performance is demonstrated with two phantoms mimicking blood vessels in small animals.

## Methods

**Figure 1:**
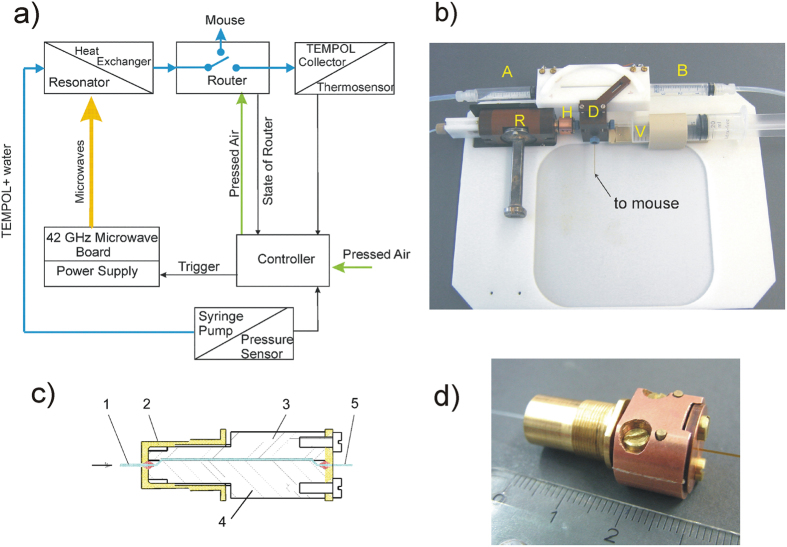
(**a**) Block-diagram of the polarizer setup. The fluid flow path is shown in blue, the microwave supply in yellow and the pressed air to drive the router in green. The upper components are situated inside the bore of the MRI magnet; the lower components are located about 2 m distant from the MRI magnet. (**b**) In-bore part of the DNP setup: microwave resonator (R); microwave waveguide (W); heat sink (H); diverter (D) with pneumatic drivers (A and B); collecting vessel for not used solution (V). (**c**) Mechanical drawing of the heat-sink design. (1) Inlet capillary ID = 0.4 mm; (2) resonator plunger; (3, 4) copper blocks; (5) outlet capillary. The arrow indicates the flow direction. The fluid pathway (370 nl dead volume) is shown in cyan. (**d**) Picture of the heat-exchanger block (copper, right side) connected to the resonator plunger (bronze, left side).

A block diagram of the overall Overhauser-DNP MRI setup is shown in Fig. 1a. The components in the upper part of the block-diagram are inside the bore of the MRI magnet. According to the “in-bore” concept, polarization transfer from electron spins to nuclei as well as MRI detection is accomplished at ambient temperature in the same 1.5 T magnetic field. The distance between the resonator and the object should be as short as possible to reduce polarization losses due to relaxation losses. For this reason, the microwave resonator was placed as close as possible to the object, where the hyperpolarized liquid should be injected. On the other hand, this distance has to be long enough to avoid MRI signal distortions by the proximity of metal parts of the resonator due to magnetic susceptibility gradients and detuning of some sections of the MRI pick-up coil. After the resonator, a heat sink cools the hyperpolarized water solution back to physiological temperature and a pneumatic diverter routes the hyperpolarized water either to the imaging object or to a collecting vessel, where the solution can be recycled and where the temperature of the flowing hyperpolarized water is measured. We used the stable TEMPOL radical as Overhauser DNP polarizing agent. Aqueous solutions with 20–40 mM concentration of TEMPOL have been prepared and filled into 20 ml syringes (Braun Melsungen, Germany) placed in a perfusion pump (ALADIN, USA) for continuous flow DNP experiments. The linewidth of each hyperfine component of the EPR spectrum of the TEMPOL solution is 3.9 G inside the MRI, the same as inside of a spectroscopic magnet. Therefore the B_0_ inhomogeneity of the MRI magnet across the 34 mm long microwave resonator did not deteriorate the saturation of the EPR line. For the magnetic field strength of our MRI scanner (Magnetom Aera 1.5 T, Siemens Erlangen Germany) microwave of 41.991 GHz was resonant with the central nitrogen (^14^N) hyperfine line of the TEMPOL radical.

The components of the DNP setup shown in the lower part of Fig. 1a are outside the bore of the MRI magnet. The 42 GHz microwave board which delivers the microwave power to the resonator (described in more detail below) is connected by means of a 2 m long WR-28 waveguide with 1 dB losses to the resonator situated inside the bore of the magnet. The DNP experiments are performed by tuning the MW frequency of the source and the resonance frequency of the microwave resonator to the EPR transitions of the TEMPOL radical, which can be monitored via the reflected microwave power by performing a frequency sweep continuous wave EPR experiment with low microwave power. After tuning the resonator to the EPR resonance frequency, a microwave amplifier with an output power of 10 W was used to saturate the EPR transition of the radical for the DNP experiments. The water/radical solution is transported by a syringe pump through Teflon tubing to the resonator.

### In-bore part of the DNP polarizer

Figure 1b depictures the in-bore part of the DNP setup. The water/radical solution flows in a 0.4 mm inner diameter quartz capillary (CMS Scientific, UK) through the MW resonator and gets thereby hyperpolarized. The sample capillary is restricted to the cylinder axis, where the electrical field component of the microwave is minimal. Nevertheless, the temperature of the solution becomes elevated within the passage due to microwave absorption. To keep the temperature of the hyperpolarized solution compatible with *in*-*vivo* physiological conditions the output of the resonator is connected to a home-build compact heat-sink (Fig. 1c,d) with the purpose to dissipate heat from liquid substrate by transferring it to the mass of copper. The heat sink consists of two almost identical copper blocks (3 and 4 in the mechanical drawing in Fig. 1c). The lower one (4) has two ports for the inlet and outlet capillaries. Both parts are glued together by means of a 30 μm thick double-sided TESA film (Krückemeyer GmbH, Germany) with a rectangular shaped microfluidic flow channel connecting the inlet to the outlet port.

**Figure 2:**
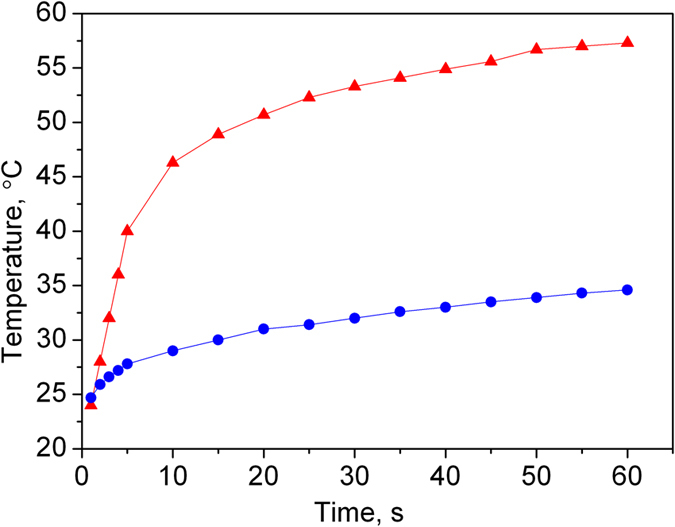
Temperature of aqueous TEMPOL solution (28 mM) with a flow rate of 1.2 ml/min after switching on the microwaves. Measurement were performed directly after the resonator (red triangles) and after the heat sink (blue circles).

Within the heat sink the fluid is in thermal contact with the copper blocks. For a flow rate of 1.2 ml/min this lowers the temperature of the hyperpolarized water from 60 °C to 30 °C within the 15–20 ms of residence time (Fig. 2). The chiller output is connected to an in-house developed fluid flow diverter (750 nl dead volume) allowing accurate timing of the injection of hyperpolarized water into the object of study after a stationary state of the hyperpolarization level and the temperature of the flowing liquid has been reached. The diverter was designed as a pneumatic 3-way stopcock device with a similar approach as presented elsewhere^25^. State of the diverter was controlled by pressed air from the controller applied to drivers A and B via two polyamide 4/6 mm tubes depending on the wanted flow direction. Two 3 ml HSW syringes have been used as pneumatic drivers. One of two outputs of the diverter is connected to the scanning phantom and the other is connected to a 20 ml syringe, which is used as the collecting reservoir for the TEMPOL/water solution. The hyperpolarized water temperature can be measured by a tiny temperature sensor (10K3MCD-1 Telemeter Electronic, Germany) placed near the position of the outlet capillary inside the Luer cannula of the syringe. The information about the current temperature as well as the state of the diverter and the total time of injection into the animal or phantom (with 0.1 sec time resolution) is displayed at the controller front panel.

All components between the resonator and the injection capillary were designed to have as small as possible dead-volumes to provide a total flow time of the hyperpolarized water from the resonator to the phantoms much shorter than the proton nuclear spin relaxation time T_1_. For this purpose all components were assembled together by tiny quartz glass capillaries (0.15 mm ID, 0.36 mm OD, Polymicro Technologies, USA) resulting in a total dead volume of only 1.2 μl. The choice of the capillary diameters was a trade-off between dead volume and back pressure to the ALADIN syringe pump with a maximum possible force of 130 N. Taking into account the dead volume of all components including the 0.15 mm capillaries the time of travel from the resonator to the object is about 50 ms at 1.5 ml/min rate resulting in a not significant loss of polarization.

### Microwave Bridge

**Figure 3:**
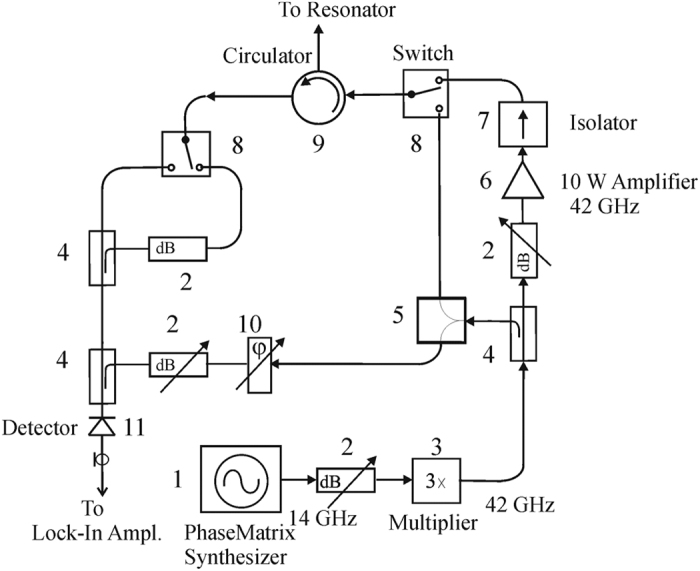
The 42 GHz microwave board: (1) microwave synthesizer; (2) attenuators; (3) frequency multiplier; (4) directional couplers; (5) power divider; (6) 10 Watt 42 GHz amplifier; (7) isolator; (8) mechanical switches; (9) circulator; (10) phase shifter; (11) detector.

Figure 3 shows the block-diagram of the microwave board, which consists of a 14 GHz synthesizer (Phase Matrix, USA), a frequency Tripler (QuinStar, USA) and a 10 W 42 GHz power amplifier (QuinStar, USA). The microwave signal reflected from the resonator can be monitored after the circulator by a microwave diode detector (Spacek Labs, USA). This signal is used to detect and tune the microwave resonator and to determine the EPR resonance frequencies for optimum DNP excitation. The output power can be regulated manually between 2 W and 11 W by a calibrated attenuator. For DNP applications microwave can be switched on or off remotely by a trigger pulse from the controller box (see Fig. 1).

**Figure 4:**
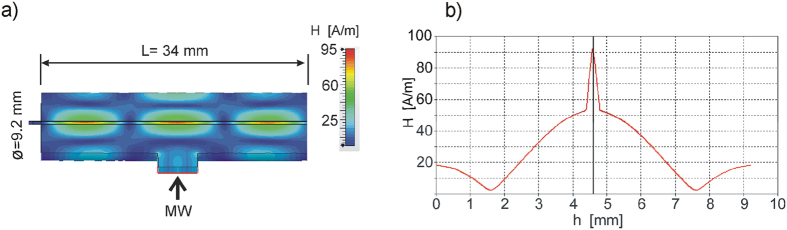
Finite element calculations of the circular TE_013_ mode resonance structure. (**a**) Distribution of the magnetic field component *H* along the central lengthwise plain of the resonator. The microwave port with the tiny iris in the wall for irradiation is shown by the arrow. (**b**) Magnetic field strength along the radial direction of the cavity. The sharp peak in the center corresponds to the capillary filled by the water. All magnetic field values are normalized to 1 Watt of incident microwave power at 42 GHz.

The most important component of the in-bore DNP polarizer is the microwave resonator. It consists of a cylindrical cavity (ID 9.2 mm) made of copper exploiting the TE_013_ circular mode. The resonator shape with the couple waveguide and the mode pattern are shown in Fig. 4a. The reason for using the TE_013_ instead of TE_011_ mode is to increase the sample volume inside the cavity. The cavity has a length of 34 mm between both plungers defined by the MW frequency of 42 GHz, resulting in a sample volume of 4.3 μl inside the cavity. The residence time inside the microwave cavity of the flowing water solution has to be on the order of the proton spin relaxation time (0.2 sec. for a 28 mM TEMPOL/water solution at 60 °C) to achieve optimum polarization enhancements. Therefore, a factor of 3 higher flow rate could be achieved with this new higher mode resonance structure compared to the TE_011_ mode resonator. The microwave is coupled to the resonator by a waveguide taper (WR-19/WR-28) through an iris (1.4 × 5.5 mm). Finite element simulations (CST STUDIO SUITE, Germany) of the cavity performance with a water-filled quartz capillary (ID 0.4 mm, OD 0.55 mm) placed along the cavity axis are shown in Fig. 4. The higher operation mode (TE_013_) results in a larger cavity and larger aqueous sample volumes with respect to TE_011_ mode cavity. However, to keep the cavity critically coupled to the waveguide the iris dimensions had to be enlarged, resulting in the lower Q- as well as conversion factor of the cavity. The calculated Q-factor of the resonator is about 110 and the conversion factor is 1.2 G/W ^½^.

### NMR detection

MRI detection was performed by a clinical 1.5 T MRI scanner (Magnetom Aera, Siemens, Erlangen, Germany) using a 8-channel surface pick-up coil (NORAS, Germany) positioned horizontally in the iso-center of the magnet bore. Phantoms were placed directly on the coil surface in the overlap of two adjacent coil elements providing the optimal RX-sensitivity. For imaging the standard 2D slice selective spoiled gradient echo (GRE) with additional inversion recovery (IR) preparation in some cases and the fast 3D GRE Volumetric Interpolated Breath-hold Experiment (VIBE) were used. The 2D GRE experiment with Cartesian k-space trajectories was performed with a repetition time (TR) of 200 ms, an echo time (TE) of 1.46 ms, a pixel bandwidth of 400 Hz, an acquisition matrix of 128 × 80 and a field-of-view (FOV) of 60 × 60 mm^2^. For the flat cell visualization the slice thickness was chosen arbitrary in a range of 20 mm to minimize TE and signal losses due to the flow induced dephasing. TR was varied in a range of 20–500 ms and excitation RF pulse flip angle (FA) from 25° to 90° to find the optimal conditions for signal contrast and sensitivity and to best observe the time-evolution of the flow pattern. The optimal MRI detection parameters were calculated to maximize SNR by using the magnetization balance approach described earlier^26^. Depending on TR single shot scans took between 1.6 and 40 seconds. The VIBE sequence is a spoiled 3D GRE sequence with TR = 50 ms, TE = 1.46 ms, FA = 20.5°, pixel bandwidth 490 Hz, with elliptical centric reordered k-space sampling (k-space center is reached after 1.1 s). The 3D data were acquired within 12.3 mm coronal slab with in-plane FOV = 63 × 63 mm^2^. The 3D acquisition matrix was 128 (read) × 83 (phase) × 22–28 (partition). By reconstruction the 2x interpolation was performed, providing a final pixel resolution of 0.52 mm^2^ × 0.6 mm. Depending on TR, pixel bandwidth and matrix amount of 3D partitions the total acquisition time was between 10 sec and 75 sec. All measurements with DNP were performed as single shot. For the reference measurement N = 8–12 averages were used to get reasonable signal intensity for signal-to-noise (SNR) calculation and DNP enhancement evaluation. The SNR was determined by measuring the signal intensity in a region-of-interest (ROI) within each phantom and the standard deviation of the noise outside the phantom. A noise profile correction procedure was applied as described in the literature^27^. All data processing was performed using in-house developed scripts for Matlab 2015a (Mathworks, Natick, USA).

## Results

Initial MRI DNP experiments with the polarizer described above were accomplished with two phantoms. The first phantom consists of a helical thin capillary mimicking blood vessels in small animals with hyperpolarized water/TEMPOL solution flowing through. The second phantom, a flat cell filled with water/TEMPOL solution in which hyperpolarized solution was injected through a thin capillary, was used to demonstrate the possibility to monitor the hyperpolarized liquid signal in larger volumes, like for example ventricles.

### 3D helical capillary phantom

**Figure 5:**
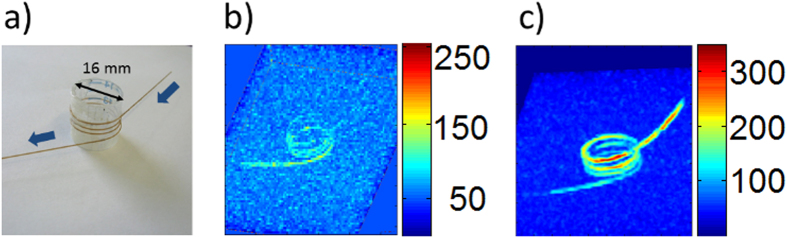
(**a**) 3D helix phantom made of a capillary with ID 0.15 mm ID/OD 0.36 mm. The blue arrows show the flow direction through the capillary. (**b**) MRI reference image of the helix without microwave (8 averages). (**c**) Single scan of the helix with 10 W of microwave power applied to the DNP resonator. All measurements are performed with the VIBE pulse sequence. The parameters are given in the text.

A 3D phantom was fabricated by using a glass capillary (ID 0.15 mm, OD 0.36 mm) wound around a PE tube (OD 16 mm) with a total capillary length of 280 mm (Fig. 5a). The inner diameter was chosen to be similar to blood vessels in small animals. With this 3D spiral we studied the potential of Overhauser DNP for 3D MRI imaging. Figure 5b shows a conventional MRI image using the VIBE sequence with the parameters described above (8 acquisitions). The liquid flows from the upper right corner to the lower left side with a flow rate of 0.9 ml/min. The water signal at the inlet is much weaker with respect to the outlet due to the larger distance of this end of the phantom to the NORAS pick-up coil used for detection. Figure 5c shows the single shot MR-image obtained with the microwaves turned on. As can be easily seen, the DNP polarized water protons lead to a strongly increased SNR and contrast of the image.

**Figure 6:**
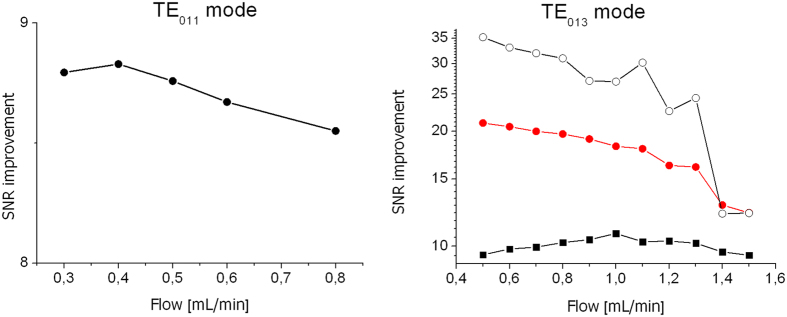
Enhancement of the SNR of images with and without microwave excitation versus the flow rate. Left side: Average enhancement achieved by the DNP method using the TE_011_ mode short microwave resonator. Right side: DNP enhancements obtained with the TE_013_ mode long microwave resonator. SNR improvements by DNP are measured at the inlet (0–5 cm, triangles), at the outlet (20–25 cm, squares) and averaged over all three turns (circles).

The SNR enhancement by the DNP method compared to the conventional MR-image without microwaves has been experimentally determined for different flow rates. The optimum flow rate for SNR improvement by DNP is a trade-off between the residence time of the liquid inside the microwave resonator and the flow time through the phantom. The first aspect requires slow flow rates to achieve maximum polarization transfer. The second aspect asks for high flow rates to reduce the relaxation losses within the flow time through the phantom. Therefore, the highest enhancement is observed on the inlet side of the phantom due to the shortest time delay after the DNP polarization process accomplished inside the microwave resonator. The largest enhancement by the DNP method averaged over the 3 turns of the phantom was 21, obtained with the TE_013_ resonator at a flow rate of 0.5 ml/min. To achieve the same SNR with a thermally polarized TEMPOL/water solution (without microwaves) a 400 times longer measurement time would be necessary. The results shown in Fig. 6 also demonstrate a 2-fold larger enhancements obtained with the longer TE_013_ resonator for a given flow rate compared to the fundamental mode TE_011_ resonator. These results have been obtained with a 28 mM TEMPOL solution, which was optimized for DNP enhancement and resulted in a proton relaxation time T_1_ = 0.2 sec.

### 2D flat cell phantom

**Figure 7:**
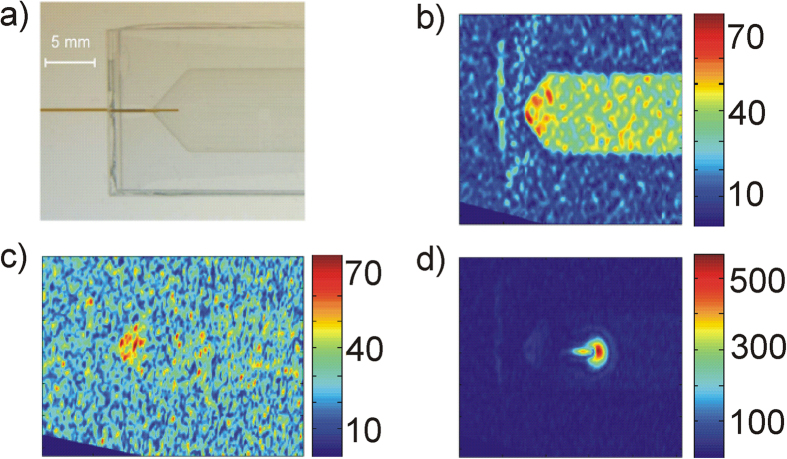
Flat cell phantom: (**a**) Photograph of the phantom. (**b**) MRI image of the 28 mM TEMPOL/water solution in the cell using a GE sequence without microwaves (8 averages). (**c**) MRI image by applying an inversion recovery filter, without microwaves (8 averages). The signal intensity close to the inlet is not inverted completely due to higher static field gradients at this position. (**d**) MRI image with the inversion recovery filter with microwave excitation. In this case the not perfectly inverted signal from the static water solution near the inlet is not visible anymore because of the much stronger DNP enhanced signal (attention should be given to the 10x higher colour bar scaling of this figure).

To demonstrate that enhanced SNR and contrast can also be obtained in larger volume samples, a second phantom was constructed where the hyperpolarized jet of liquid is injected into a flat cell with an extended volume filled with the TEMPOL/water solution. Figure 7a shows the flat cell phantom made of two Plexiglas plates with a separation of 0.4 mm. The hyperpolarized solution flows through the capillary (brown part in Fig. 7a, ID 0.15 mm) before injection into the flat cell. The outlet of the flat cell (at the right side of Fig. 7a) is open so that the flowing liquid can leave the cell. MRI images of the flat cell phantom containing a 28 mM TEMPOL aqueous solution have been obtained using a GRE pulse sequence. Images were interpolated from 0.35 mm × 0.6 mm pixels over a 30 mm × 30 mm FOV. The slice thickness was set to 12 mm and the total acquisition time was 75 s per image. A reference image without microwave was acquired with a conventional 2D GRE sequence (8 averages of acquisition, Fig. 7b). The TEMPOL/water solution in the capillary is not visible because of the fast flow rate of 0.9 ml/min. Figure 7c shows the image obtained by applying an inversion recovery sequence (still without microwave excitation). The inversion time delay (IT = 120 ms) was adjusted to provide maximal cancelation of the Boltzmann polarized background signal^26^, therefore hardly any signal and contrast is left. Figure 7d finally depictures the MRI image obtained with 10 W of microwave excitation (single shot acquisition). Note the almost factor of 10 different color-coding of the signal intensity in this figure, demonstrating impressively the amount of hyperpolarization obtained by the Overhauser DNP mechanism. Because the hyperpolarized NMR signal is much larger and has opposite sign compared to the Boltzmann thermal equilibrium signal the injected jet can be detected with very high contrast inside the non-polarized water pool.

**Figure 8:**
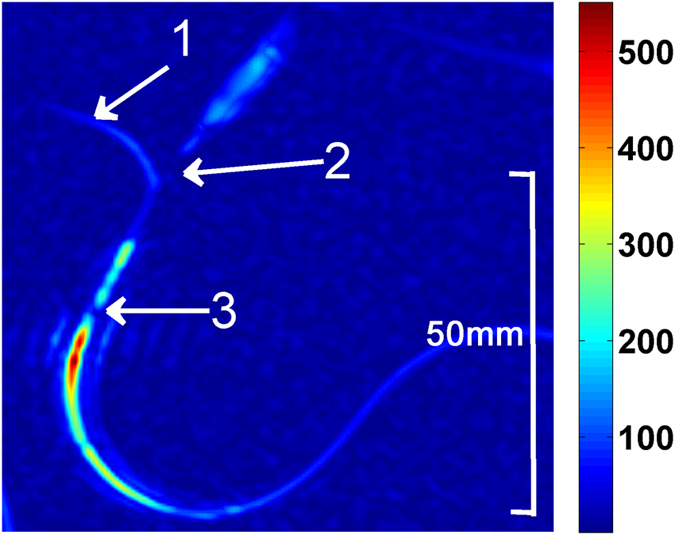
Phantom for injection of DNP enhanced liquid in a blood vessel: (1) 1 mm ID plastic tubing filled with 0.9% NaCl physiological buffer solution with a flow rate of 2.4 ml/min. (2) Quartz capillary with 0.15 mm ID used for the injection of 28 mM TEMPOL solution with a flow rate of 1.5 ml/min. (3) End of the injection capillary inside the phantom blood vessel. The injected hyperpolarized liquid is clearly visible with enhanced contrast in the non-polarized physiological buffer flow stream.

First experiments on a blood vessel phantom demonstrate that even under dilution of the injected hyperpolarized liquid in a bloodstream of an animal, hyperpolarized signal can be obtained with our existing setup (Fig. 8). The used phantom consist of a 1 mm ID plastic tubing connected to a perfusion pump with a physiological buffer solution with 2.4 ml/min flow rate, corresponding to the aorta of a mouse. The hyperpolarized water/TEMPOL solution was injected into this stream by a 0.15 mm ID quartz capillary with a flow rate of 1.5 ml/min. Imaging in this vessel phantom featured a contrast enhancement up to a factor 5, which decreased by 50% after 3.5 cm and went back to its origin after 5 cm during dilution by the downstream.

## Discussion

Here we describe a new experimental approach and setup to hyperpolarize liquid water proton signals for MRI applications at 1.5 T magnetic field. Polarization transfer from unpaired electron spins of radicals to the proton nuclear spins is achieved by microwave irradiation of the TEMPOL/water solution directly inside the bore of the MRI magnet. This ‘in-bore’ approach is different from other approaches which hyperpolarize the proton signal outside the MRI magnet at lower magnetic field values. The advantage of our approach is that no additional magnet is required and the polarization loss arising from the transfer of the hyperpolarized liquid from the lower polarizing to the higher observing field is avoided. Additionally the distance to the object under study can be minimized in this new setup. On the other side higher frequency microwave equipment operating at 42 GHz is needed for the excitation of the radical in the higher field of 1.5 T, where the Overhauser polarization transfer mechanism also becomes less efficient. Nevertheless, by using a tailored microwave resonance structure with an efficient excitation of the electron spin transitions of the radical DNP enhancements up to −100 could be reached^24^. In combination with a heat sink directly after the DNP resonator high nuclear spin polarizations can be reached under continuous flow conditions without overheating the liquid solution.

With the prolonged TE_013_ mode microwave resonator in combination with a 10 W microwave amplifier up to 1.5 ml/min of hyperpolarized water solution could be delivered continuously. With a capillary phantom we could demonstrate that this leads to an overall SNR increase of 12 for 3D MR images along a 28 cm capillary path length (ID 0.15 mm). This corresponds to a 144-fold decrease in measurement time or factor 2.3 smaller isotropic voxel at a given SNR, opening up the possibility to monitor very fast dynamical processes. Furthermore, we demonstrated with a second phantom that also the injection of hyperpolarized liquid in more bulky cavities can be observed with strongly increased sensitivity and contrast. Of course the ideal laminar flow in our capillary phantom might not be simply transferred to an *in*-*vivo* vascular system suffering from pulsation, varying diameters, angles and branches, which is topic of further studies.

The maximum achievable SNR enhancement of the OE-DNP method under flow conditions is a complicated function of the flow rate. On the one hand the flow rate should not be too fast to allow maximum DNP polarization build up inside the microwave resonator, which is on the time scale of the nuclear spin relaxation time. For the 25 mM TEMPOL/water solution the longitudinal proton relaxation time is about 0.2 s. Additionally, the residence time of the liquid inside the resonator is proportional to the amount of microwave heating of the TEMPOL/water solution. Because the Overhauser polarization transfer efficiency at a magnetic field of 1.5 T strongly increases by raising the temperature^28^ higher DNP polarizations can be achieved under such conditions. The heat-exchanger installed directly after the resonator is capable to cool down liquid temperature below 35 °C during a long-time microwave excitation. Depending on the flow rate its heat capacity must be taken into account complicating thermal management during *in*-*vivo* imaging. This issue is addressed by our live temperature measurement sensor. The risk of a volume overload has been addressed with the pneumatic flow diverter. Furthermore it keeps the dead volume low. On the other hand a low flow rate has obvious disadvantages for MRI imaging applications. A small amount of hyperpolarized solvent available per time will be strongly diluted after injection into the blood stream of living objects reducing the maximum obtainable SNR enhancement by the DNP method. Additionally the same volume of hyperpolarized liquid will be exposed to multiple RF-pulses and phase encoding cycles of the MRI experiment. For the VIBE MRI experiments used with our 3D helical capillary phantom the repetition time TR was 50 ms and the total liquid volume 4.9 μl. To have identical starting conditions (freshly hyperpolarized liquid) for each phase encoding step a flow rate higher than 6 ml/min would be required, much larger than the maximum flow rate of 1.5 ml/s achievable with our current setup. Therefore, the hyperpolarized magnetization is driven in the transient-state regime^26^. However, differently from samples at thermal equilibrium polarization the magnetization of hyperpolarized samples does not relax back to the initial value after the RF pulse but relaxes to the much lower Boltzmann equilibrium. Therefore, pulse sequences for ^1^H MRI with continuous-flow injection of DNP hyperpolarized liquid should use hybrid approaches combining dynamic MRI techniques developed for transient state Boltzmann magnetization and fast sampling techniques used for hyperpolarized magnetization in batch mode^29^,^30^,^31^,^32^,^33^,^34^. Even without the RF pulses applied the hyperpolarized sample will relax back to the Boltzmann equilibrium.

The opposite polarity of the Overhauser DNP signal allows to apply an inversion recovery filter, which suppresses the signal from non-polarized water protons and allows detection with very high contrast of only the injected hyperpolarized water jet^26^. This method can be used to investigate flow dynamics and perfusion at specific target surfaces with enhanced spatial and temporal resolution.

Furthermore, the parts of the phantom further away from the microwave resonator will exhibit lower signal enhancements, complicating image reconstruction of hyperpolarized flowing liquids. The last aspect could of course also be considered as an advantage because the signal intensity somehow encodes the time passed after the hyperpolarization event. In any case methodological developments on the MRI pulse sequence side could further improve the SNR gain obtained by the Overhauser DNP method. A good starting point for further optimization could be fast dynamic sequences with non-uniform sub-Nyquist k-space sampling. These sequences, in particular the so-called ‘Golden Angle Radial’ type of experiments^35^, are designed to use minimum amount of phase encoding lines of k-space for image reconstruction exploring image information redundancy. This allows to minimize the magnetization losses by relaxation due to shorter measurement time and to increase the flip angle per excitation providing additional SNR increase. For optimizing the density of the k-space coverage by radial spokes the hyperpolarization pool created by DNP could be helpful for both maximizing the SNR and for reducing reconstruction artefacts due to under-sampling.

Here, we could show that already with existing standard MRI pulse sequences used for routine applications SNR enhancements of more than 10 could be easily reached by the OE-DNP hyperpolarization method. For our maximum flow rate of 1.5 ml/min the hyperpolarization loss at the end of the 280 mm capillary of our 3D helical phantom was about 28% due to the application of repetitive RF pulses and 58% due to T_1_ relaxation losses for a laminar flow. The losses due to the short relaxation time induced by the paramagnetic radicals could be removed by immobilizing the TEMPOL radical inside the DNP resonator^36^,^37^,^38^,^39^. This would not only allow observing the hyperpolarized liquid pathway inside the blood vessels for a prolonged time and length after the injection point but also would avoid the exposure of the object to the radical. Despite the fact that such kind of radicals has been already used for preclinical *in*-*vivo* MRI applications^40^, the perspective to inject pure hyperpolarized water is very attractive. This observed contrast can be further improved by using inversion recovery and optimization of acquisition parameters. In principle, also the flow rate can be further improved by more elaborate oversized or parallelized resonance structures combined with high power microwave amplifiers.

In the future we will explore the amount of hyperpolarization that we can achieve with our current setup in MR-aortography, cerebral angiography and parenchymal perfusion. Of course there are general challenges for *in*-*vivo* applications like catheterization near to the region of interest in order to achieve a sharp contrast peak and avoid spatial signal loss and acquisition artefacts by ventilation and cardiac pulsation. Besides, animal experiments face potential challenges in homeostasis of temperature and electrolytes and in the surveillance of vital functions, which need to be addressed in any case.

By the results achieves on phantoms, we demonstrated the performance of our in-bore DNP setup, which is capable of flow rates and signal enhancements promising for small animal experiments, thus depicting it as a very interesting new accessory to a standard 1.5 T scanner for specific proton MRI applications.

## Additional Information

**How to cite this article:** Denysenkov, V. *et al*. Continuous-flow DNP polarizer for MRI applications at 1.5 T. *Sci. Rep.* **7**, 44010; doi: 10.1038/srep44010 (2017).

**Publisher's note:** Springer Nature remains neutral with regard to jurisdictional claims in published maps and institutional affiliations.
